# DeepMethylation: A deep learning framework for tissue-specific DNA methylation prediction and functional variant annotation

**DOI:** 10.1371/journal.pcbi.1014476

**Published:** 2026-07-01

**Authors:** Wenran Li, Shijia Yu, Yingyu Cheng, Sijia Wang

**Affiliations:** Shanghai Institute of Nutrition and Health, University of Chinese Academy of Sciences, Chinese Academy of Sciences, Shanghai, China; Burnet Institute, AUSTRALIA

## Abstract

DNA methylation is a key epigenetic modification that regulates gene expression and plays a vital role in cell differentiation, development, and tumorigenesis. However, large-scale experimental profiling of genome-wide DNA methylation remains time-consuming and limited in coverage. We present DeepMethylation, a deep learning framework that integrates DNA sequence and tissue-specific epigenomic features to predict CpG methylation status across the genome. DeepMethylation achieves state-of-the-art performance (average AUROC 0.909) across tissues, accurately imputes methylation beyond array-covered sites, and enables robust extension from 450k to EPIC array coverage. Feature importance analysis revealed consistent patterns of epigenomic feature contributions across tissues. We also introduced Delta DeepMethylation (DDM), a variant evaluation model to estimate the epigenetic effects of SNPs on DNA methylation. DDM-predicted variant effects were consistent with methylation quantitative trait loci (mQTLs) and not confounded by linkage disequilibrium (LD). Our framework provides a powerful tool for genome-wide methylation prediction and regulatory variant interpretation across tissues.

## Author summary

In this study, we set out to address a key challenge in studying DNA methylation, a chemical mark on DNA that helps regulate genes and plays an important role in development, aging, and disease. Measuring methylation across the whole genome is expensive and incomplete, leaving gaps in our understanding. To overcome this, we built DeepMethylation, a deep learning model that predicts methylation patterns across the genome by combining DNA sequence information with features that are specific to each tissue. With this tool, we can extend the value of existing data, fill in missing information, and make accurate predictions even in regions not directly measured by current technologies. We also developed a companion model that estimates how small changes in DNA can influence methylation, offering new insights into how genetic differences may affect health and disease. By making these tools freely available, we aim to provide researchers with a cost-effective way to study epigenetic regulation and to help guide discoveries in human biology and medicine.

## Introduction

Epigenetics refers to the inheritance of variations in gene activity or chromatin states without changes to the underlying DNA sequence, with DNA methylation being one of the most extensively studied epigenetic modifications [1]. Specifically, 5-methylcytosine (5mC) plays a crucial role in gene transcription and expression, chromatin stability, and genomic imprinting, significantly impacting development, aging, tumorigenesis [2,3]. Recent advancements have highlighted the importance of DNA methylation in the diagnostic classification and prognostic assessment of tumors [4–7]. Furthermore, growing research has explored the reprogramming of CpG methylation patterns and the inheritance of methylation marks in mammalian genomes during embryonic development [8,9]. These studies all rely on a comprehensive understanding of DNA methylation levels; therefore, accurate measurement of genome-wide DNA methylation remains essential for advancing research in this field.

In the past decades, the most widely used methylation profiling technology is the Infinium MethylationEPIC BeadChip (EPIC/850K), which measures DNA methylation levels of approximate 850,000 preselected CpG sites across the genome. However, the EPIC array assay only covers 3% of all CpG sites in the entire genome, focusing primarily on CpG islands, promoter regions, coding regions, open chromatin, and enhancer regions [10]. In comparison, whole-genome bisulfite sequencing (WGBS) quantifies DNA methylation levels at about 26 million CpG sites in the human genome. However, limited by the sequencing design, WGBS is subject to incomplete bisulfite conversion and exhibits low coverage in regions with high cytosine content, such as CpG islands. Additionally, the high cost of WGBS makes it impractical for large-scale sample analysis [11]. Therefore, there is a clear need for computational models to enable convenient and cost-effective prediction of genome-wide DNA methylation.

Recently, Zhang *et al.* utilized ~300 features including neighbor CpG sites information, genomic position, DNA sequence properties, and DNA regulatory elements, employing a random forest (RF) classifier [12]. Liu *et al.* used pseudo trinucleotide composition with a support vector machine (SVM)-based classifier called iDNA-Methyl [13]. Zheng *et al.* used DNA composition features, conversed transcription factor binding sites (TFBSs), general attributes and physicochemical properties, conservation, and diversity of the selected region with a support vector machine (SVM)-based classifier called CpGIMethPred [14]. Despite their comprehensive feature sets, these methods were usually unable to capture long-range dependencies of sequence and some of them were only suitable for the prediction of specific regions of the genome, limited by the differential methylation patterns of CpG sites in different regions [12–24].

With the development of deep learning technologies, methods that capture local sequence information directly from raw DNA sequences have been proposed to predict the methylation levels of CpG sites [25–31]. Angermueller *et al.* proposed DeepCpG, a computational method based on deep neural networks that integrated DNA sequence patterns and methylation states of neighboring CpG sites for predicting single-cell methylation states [26]. Tian *et al.* introduced MRCNN, a convolution neural network (CNN)-based model [27], and Zhou *et al.* designed INTERACT, an integrated CNN and transformer model [31], to predict genome-wide DNA methylation states using DNA sequence feature. However, these methods primarily relied on DNA sequence data and did not incorporate genomic features, which limited their model performance and ability in tissue-specific prediction. Therefore, a more robust deep learning model that integrates both sequence information and genomic features is required to improve performance and enable accurate whole-genome prediction.

Here, we presented a deep learning model named DeepMethylation, designed to predict DNA methylation status of genome-wide CpG sites by integrating local DNA sequences and tissue-specific epigenomic annotations. We demonstrate that DeepMethylation achieves state-of-the-art performance in predicting DNA methylation status, offering accurate and robust genome-wide predictions across various tissues. Feature importance analysis reveals both the consistency and variability of epigenomic feature relevance across different tissues. Additionally, we developed a variant-evaluation model, Delta DeepMethylation (DDM), to assess the epigenetic effect of genetic variants on DNA methylation levels. When compared to methylation quantitative trait loci (mQTL) association analysis, we found that the genetic effects predicted by DDM showed a consistent trend with those observed in mQTLs, while our predicted genetic effects were not confounded by LD.

## Results

### DeepMethylation achieves state-of-the-art performance in predicting DNA methylation status

**Fig 1:**
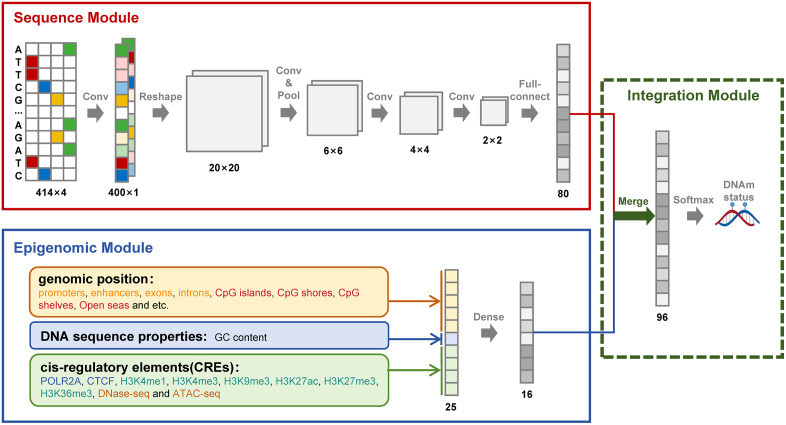
Design of the DeepMethylation model.

We developed a deep-learning model, DeepMethylation, that integrates local DNA sequence information with tissue-specific epigenomic annotations to predict the methylation status of CpG sites. Specifically, the model takes as input one-hot encoded DNA sequences surrounding the target CpG site together with epigenomic annotations at the corresponding genomic location (S1-S2 Tables; S1 Data). DeepMethylation consists of three modules: (i) a CNN-based sequence module for capturing local DNA sequence features, (ii) an MLP-based epigenomic module for extracting epigenomic information around the CpG site, and (iii) an integration module that combines these features to predict CpG methylation status (Fig 1).

**Fig 2:**
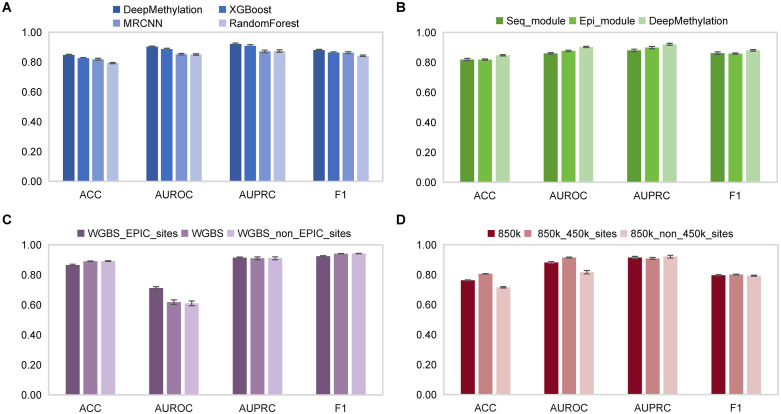
Prediction of DNA methylation status from DNA sequences and epigenomic annotations. **(A)** Comparison of model performance in predicting DNA methylation status of independent CpG sites in blood tissue. Each model predicts CpG methylation status across individual samples, with bar heights and error bars representing the mean and standard deviation of each evaluation metric across test samples. **(B)** Comparison of the predictive contribution between the sequence module and the epigenomic module. **(C)** External validation of DeepMethylation using WGBS data in blood tissue. CpG sites in each WGBS sample are classified as either EPIC sites (covered by the EPIC array) or non-EPIC sites. Models trained on EPIC-array samples are applied to WGBS samples for validation. **(D)** Extension of 450k methylation data to EPIC methylation data in blood tissue. CpG sites in the 450k array are categorized as either 450k sites (covered by the 450k array) or non-450k sites. Models trained on 450k-array samples are tested on EPIC-array samples.

To evaluate whether DeepMethylation can learn informative features for DNA methylation prediction, we trained and tested the model using 754,119 CpG methylation measurements from the Illumina EPIC array in blood tissue [32]. DeepMethylation achieved an average accuracy of 0.847 and an average AUROC of 0.903, outperforming the state-of-the-art model MRCNN [27], another CNN-based model, with an average accuracy of 0.819 and an average AUROC of 0.852. DeepMethylation also outperformed two additional baseline models using the same input (Fig 2A).

To assess the contribution of each module, we compared the full model with models using sequence features alone or epigenomic features alone. DeepMethylation integrating both sequence and epigenomic annotations achieved significantly better performance than models using only a single input modality. Moreover, the epigenomic module contributed more strongly than the sequence module to DNA methylation prediction (Fig 2B). We also evaluated the contribution of neighboring CpG information by incorporating epigenomic features from the nearest CpG site into the model, which resulted in only marginal performance improvement (S1 Text and S1 Fig).

To further assess whether DeepMethylation can capture continuous methylation variation, we performed an additional regression-based analysis on the same EPIC array dataset used for model training and evaluation. The regression model achieved a Pearson’s correlation coefficient (PCC) of 0.76 and an R^2^ of 0.58 (MAE = 0.20, MSE = 0.07), indicating that DeepMethylation can effectively predict continuous methylation levels (S2A Fig). We further compared the regression performance of DeepMethylation with that of MRCNN under the same setting. DeepMethylation outperformed MRCNN (MRCNN: PCC = 0.69; R^2^ = 0.47), demonstrating improved performance in modeling continuous methylation signals (S2B Fig).

### DeepMethylation enables genome-wide prediction of DNA methylation beyond array coverage

We applied the DeepMethylation model to predict DNA methylation status across the entire genome. To assess the accuracy of the whole-genome prediction, we collected 36 WGBS samples of 14 primary cell types in blood tissue [17], yielding high-quality methylation data for approximately 24 million CpG sites after quality control. Each CpG site was classified as either covered by the EPIC array (EPIC CpG sites) or unique to the WGBS dataset (non-EPIC CpG sites).

Using the blood-specific DeepMethylation model trained on EPIC array data, we predicted methylation status for the ~ 24 million CpG sites and evaluated its performance against true WGBS-derived methylation values. Across all CpG sites, the model achieved an average accuracy of 0.890 and an average AUROC of 0.618 (Fig 2C). Performance on non-EPIC CpG sites was similar to the genome-wide average, whereas EPIC CpG sites showed higher discrimination performance, with an AUROC of 0.712.

To further evaluate performance on continuous methylation values, we applied a regression-based framework to WGBS data using the model trained on EPIC array data. Predicted methylation levels were then compared with observed WGBS-derived values. We observed modest agreement overall (R^2^ = 0.20), with higher concordance at EPIC CpG sites (R^2^ = 0.42) than at non-EPIC CpG sites (R^2^ = 0.17) (S3 Fig). These results are consistent with the performance gap observed under the binary formulation and further indicate that methylation prediction is more challenging at non-EPIC CpG sites.

### Cross-platform imputation from 450k to EPIC array data

Although the EPIC array expanded the coverage of DNA methylation profiling from 450k to over 850k CpG sites, a large volume of existing methylation data was generated using the earlier 450k platform. To bridge this gap, we applied DeepMethylation to impute unmeasured CpG sites and extend 450k data to EPIC-level resolution. Specifically, we trained DeepMethylation on 450k array data and evaluated its performance on an independent EPIC dataset. To assess its extrapolation capability, the EPIC test set was divided into two subsets: CpG sites shared with the 450k array (450k sites) and those unique to the EPIC array (non-450k sites).

DeepMethylation, trained solely on 450k data, achieved an average accuracy of 0.762 and an AUROC of 0.882 on the full EPIC test set, performing only slightly below a model trained directly on EPIC data (Fig 2D). Importantly, the model maintained strong performance on non-450k CpG sites, with an accuracy of 0.716 and an AUROC of 0.817, demonstrating its ability to generalize beyond the 450k platform. As expected, the performance on 450k CpG sites slightly exceeded that on the full EPIC set. These results highlight DeepMethylation’s potential to enhance and reinterpret legacy 450k datasets, expanding their utility for downstream analyses and cross-platform comparisons.

### Tissue-specific and cross-tissue performance of DeepMethylation

**Fig 3:**
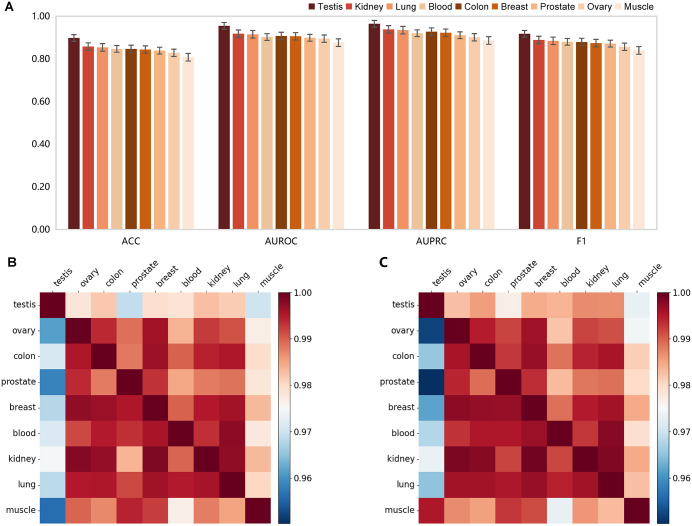
Prediction of DNA methylation status across multiple tissues. **(A)** Predictive performance of each tissue-specific DeepMethylation model. Each model predicts DNA methylation status for independent CpG sites in samples from the same tissue used for training. Bar heights represent the mean of evaluation metrics (e.g., AUROC, AUPRC), and error bars indicate the standard deviation across testing samples. **(B)** Relative AUROC of each tissue when tested using different tissue-specific DeepMethylation models. The horizontal axis indicates the training tissue, and the vertical axis indicates the testing tissue. Each cell value is normalized by the diagonal value of its row, reflecting the relative AUROC of a tissue in cross-tissue models compared to its own model. **(C)** Relative AUPRC of each tissue tested in different tissue-specific DeepMethylation models.

To evaluate the ability of DeepMethylation to capture tissue-specific features underlying DNA methylation patterns, we applied the model to nine distinct tissues (transverse colon, ovary, prostate gland, testis, kidney, lung, breast, skeletal muscle, and whole blood) and compared their tissue-specific prediction performance. Overall, DeepMethylation achieved consistently high performance across tissues, with an average accuracy of 0.847 and an average AUROC of 0.909, indicating its capacity to learn and generalize tissue-specific methylation features (Fig 3A; S2 Data). Notably, the model performed best in testis tissue, reaching an accuracy of 0.898 and an AUROC of 0.955.

We next assessed the model’s generalizability across tissues by performing cross-tissue prediction: models trained in one tissue were used to predict DNA methylation in the remaining eight tissues, resulting in 81 (9 × 9) pairs of tissue comparisons. DeepMethylation maintained reliable cross-tissue performance, with AUROC and AUPRC values exceeding 0.830 across all pairs (Fig 3B-3C). As expected, the highest prediction accuracy was consistently observed when training and testing were performed on the same tissue (S3 Table), aligning with findings from previous studies [23]. These results support the feasibility of cross-tissue methylation prediction, while also highlighting the dependency of predictive performance on specific tissue combinations.

### DeepMethylation captures consistent predictive features across different tissues

**Fig 4:**
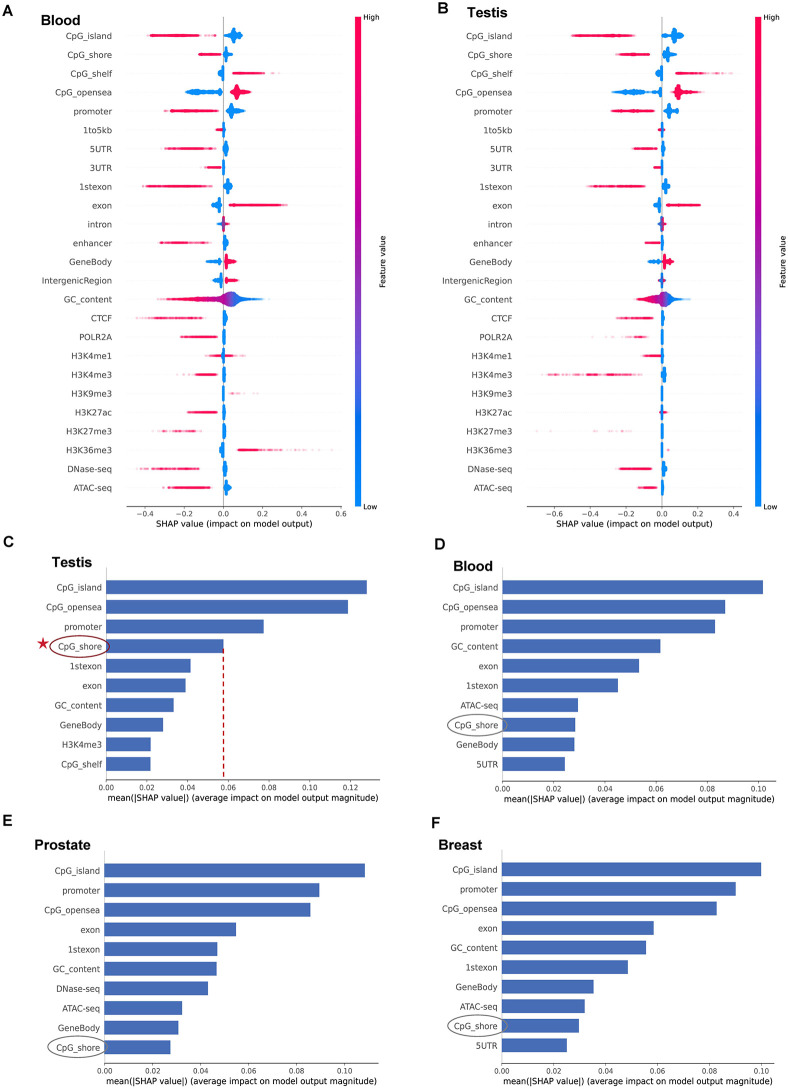
Importance of 25 epigenomic features across multiple tissues as evaluated by SHAP values. **(A-B)** SHAP summary plots showing the contribution of 25 epigenomic features to DNA methylation predictions in blood (A) and testis **(B)**. Higher SHAP values indicate a stronger positive influence on predicted DNA methylation levels. Feature values are color-coded, with red indicating higher values and blue indicating lower values. **(C-F)** Bar plots showing the top 10 most important epigenomic features ranked by mean absolute SHAP values (|SHAP value|) in testis **(C)**, blood **(D)**, prostate **(E)**, and breast **(F)**. The red star highlights the CpG shore feature in testis tissue, indicating its distinct contribution compared to other features.

To investigate the impact of epigenomic features on prediction performance, we applied SHAP (SHapley Additive exPlanations) [33] to systematically quantify the contribution of each input feature in the DeepMethylation model. The absolute SHAP values indicate the magnitude of feature contribution to the prediction, while their sign reflects the direction of the effect. We computed SHAP values for each of the 25 features in the epigenomic module—including genomic positions, DNA sequence properties, and cis-regulatory elements (CREs)—across nine different tissues. The analysis revealed consistent feature contribution patterns across tissues (Fig 4A-4B). Notably, CGI and CpG opensea emerged as the most influential features. CGI was negatively associated with DNA methylation, while CpG opensea showed a positive correlation. These findings are in line with previous studies showing that over 90% of CpG sites in the human genome are hypermethylated, whereas CGI regions tend to be hypomethylated [34]. Other features, including promoter regions, exons, GC content, and chromatin accessibility, were also negatively correlated with DNA methylation levels—likely because CGIs are often located within promoters and exons, where hypomethylation is functionally necessary [35,36].

We next calculated the average absolute SHAP values for each feature to better visualize their overall contribution across tissues (Fig 4C-4F). Among genomic position features, CGI, CpG opensea, and promoter regions ranked as the top contributors. In the DNA sequence property group, GC content consistently showed high importance. For CRE features, DNase-seq and ATAC-seq signals were the most informative, while transcription factor binding and histone marks generally had lower contributions. Interestingly, the CpG shore region showed markedly higher importance in testis compared to other tissues. This raises the possibility that the superior predictive performance of DeepMethylation in testis may be linked to the distinct distribution or regulatory role of CpG shores in this tissue. Despite their potential relevance, the biological functions of CpG shores remain incompletely understood [37], and tissue-specific differences—particularly in testis—have been rarely explored in the literature.

### DeepMethylation identifies variants with potential regulatory impact on DNA methylation

**Fig 5:**
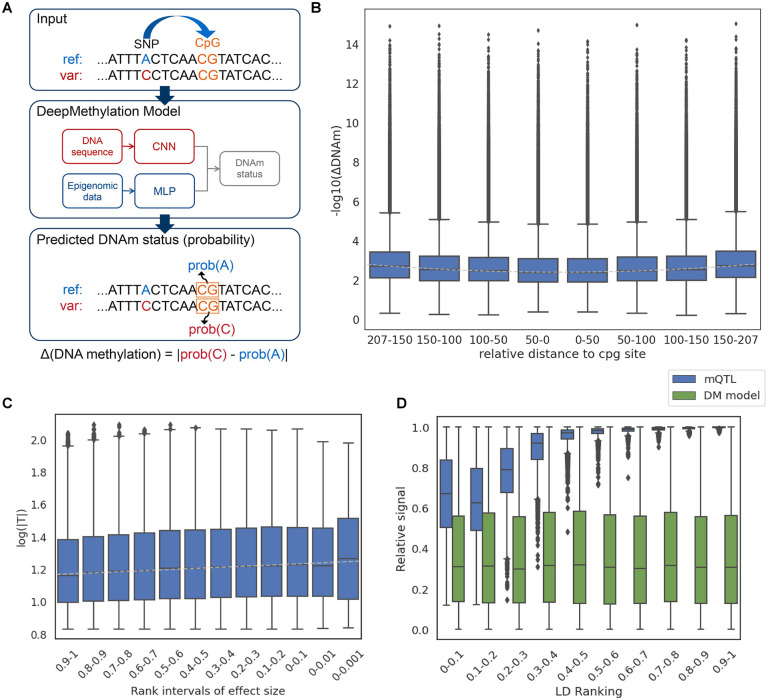
Computation, characterization, and validation of predicted genetic effects. **(A)** Schematic overview of the Delta DeepMethylation (DDM) model, which computes predicted genetic effects and identifies DNA methylation regulatory variants. **(B)** Relationship between predicted genetic effects and SNP-CpG distance. Variants are grouped by their relative distance to CpG sites (x-axis), and the box plot shows the distribution of predicted genetic effects (y-axis) in a representative blood sample using the blood-specific model. **(C)** Comparison of variant signals derived from association analysis vs. DDM predictions, plotted against LD with the top variant per CpG site. For each CpG site, the relative signal is calculated as the signal of a variant divided by that of the top variant. Association signals are represented by log₁₀(|T value|), while DDM signals are measured by the absolute predicted genetic effect. **(D)** Comparison between DDM-predicted SNP effects and empirical mQTL signals. SNP-CpG pairs are ranked by their predicted genetic effects in each training sample of the corresponding tissue. The average mQTL signal (y-axis) is plotted for each rank interval (x-axis), where the “0-0.001” interval represents the top 0.1% of SNP-CpG pairs with the largest predicted effects.

Leveraging DeepMethylation’s ability to capture base-pair level sequence features, we developed a variant evaluation framework, termed DDM, to predict the genetic effects of SNPs on CpG methylation levels (Fig 5A). By inputting both the reference and alternative allele sequences surrounding a CpG site, DDM calculates the difference in predicted methylation levels, thereby estimating the regulatory impact of each SNP on DNA methylation.

To characterize the variant effects predicted by DDM, we first examined the relationship between the distance of SNPs to CpG sites and their predicted effect sizes. We found that variants closer to CpG sites tended to exhibit larger predicted genetic effect sizes, consistent with the spatial pattern observed for mQTLs derived from genetic association analysis (Fig 5B). Then, we utilized mQTLs derived from genetic association analysis [38] to assess the concordance between DDM predictions and empirical regulatory signals. Specifically, we computed association signals for all SNP-CpG pairs where the SNP resided within a 414-bp window of EPIC array CpGs. When stratified by predicted effect size, SNP-CpG pairs with larger predicted effects exhibited stronger mQTL signals (i.e., higher absolute significance values), supporting the consistency between DDM predictions and empirical mQTL data (Fig 5C). We further performed a direct comparison between DDM-predicted effects and mQTL-derived effect estimates for matched SNP-CpG pairs. Overall, a statistically significant trend was observed (S4 Fig), indicating that DDM captures part of the regulatory signal reflected by mQTLs.

Next, we examined whether DDM-predicted effects were influenced by linkage disequilibrium (LD), as traditional mQTL association signals can be affected by LD structure. Whereas mQTL association strength increased with LD to the lead SNP, DDM-predicted effect sizes showed no significant correlation with LD strength (Fig 5D). This indicated that, unlike traditional mQTLs, the variant effects predicted by our model were less influenced by LD structure and thus offer a more direct assessment of the potential regulatory effects of variants with reduced influence from LD. In addition, we benchmarked DDM against DeepSEA2.0 (Beluga) [39,40], a representative sequence-based regulatory variant prediction framework. Using mQTL-defined positive and negative SNPs, we found that both DDM and its sequence-only version (DDM_seq) achieved higher AUROC than the Beluga-based aggregation scores, with the full DDM model showing the best performance (S2 Text and S5 Fig). We further performed a sensitivity analysis to examine the impact of perturbations in epigenomic features on variant effect prediction. Predicted effects remained stable under moderate perturbations, supporting the robustness of DDM to moderate variation in epigenomic inputs (S3 Text and S6 Fig).

### Co-methylation patterns influence DNA methylation stability and genetic susceptibility

**Fig 6:**
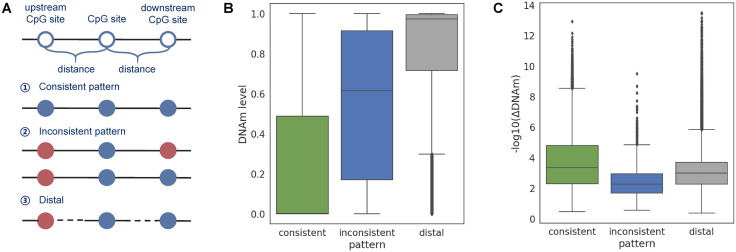
Influence of co-methylation patterns on DNA methylation levels and predicted genetic effects of CpG sites. **(A)** Definition of three co-methylation patterns based on the methylation status of neighboring CpG sites. For each target CpG site, neighboring CpGs located within a specified distance (D = 207 bp) upstream and downstream are examined. If no neighboring CpGs are found within this range, the site is classified as “distal.” If neighboring CpGs are present, the site is categorized as “consistent” or “inconsistent” depending on whether their methylation status matches that of the target CpG site. **(B)** Comparison of predicted DNA methylation levels among CpG sites in the three co-methylation patterns. **(C)** Comparison of predicted genetic effects on DNA methylation levels across the three co-methylation patterns.

DeepMethylation provides genome-wide predictions of DNA methylation levels, yielding a sufficient number of informative CpG sites to investigate patterns among different CpG sites. Based on the predicted methylation levels across the genome, we defined a set of co-methylation patterns to explore how neighboring CpG sites influence the methylation status of a target site. Specifically, we classified CpG sites into three distinct patterns according to the distance to their neighboring CpG sites and the consistency of their methylation status (Fig 6A). We then calculated the predicted methylation probability for each CpG site under these different patterns. Our analysis revealed that CpG sites that were close to neighboring sites and shared a consistent methylation status tended to be hypomethylated. In contrast, those with inconsistent methylation status were more likely to be hypermethylated. Moreover, CpG sites located far from their neighbors also tended to be hypermethylated (Fig 6B). These observations are consistent with previous studies reporting that transcription factors (TFs) often bind to continuous genomic segments, leading to co-hypomethylation of all CpG sites within the binding region, while CpG sites in opensea regions are predominantly hypermethylated.

Furthermore, we computed the predicted genetic effects of variants on DNA methylation status of CpG sites with three different patterns. We found that CpG sites with inconsistent patterns were more affected by SNP than those with consistent pattern (Fig 6C), which indicated that the methylation of CpG sites in a co-methylation status was more stable and less susceptible to variants.

## Materials and methods

### Data collection and preprocessing

The Illumina EPIC Methylation array data for nine tissues (breast, kidney, colon, lung, muscle, ovary, prostate, testis, and whole blood) were obtained from the GTEx project, as published in the study conducted by Oliva et al [32]. The dataset generated DNA methylation profiles of 754,119 CpG sites for 987 samples from GTEx v8 cohort. We aligned CpG sites detected across all samples from all tissue types and retained only commonly detected sites with comprehensive annotations. Genome sequences were derived from the UCSC hg19, GRCh37 (Genome Reference Consortium Human Reference 37) with GenBank assembly accession number GCA_000001405.1 [41].

A comprehensive list of 25 epigenomic features were used in prediction (S1 Table). The genomic position, including gene coding region category (promoters, enhancers, exons, first exons, introns, 3’ UTRs, 5’ UTRs, 1–5 kb region, gene body and intergenic region) and location in CGIs (CpG islands, CpG shores, CpG shelves and Open seas), were obtained from annotatr, a R package for annotating genomic region [42]. The GC content was calculated based on 414 bp DNA sequences around the CpG sites. The cis-regulatory elements (CREs) included 2 specific transcription factor binding sites (TFBSs; POLR2A and CTCF), 6 histone modification marks (H3K4me1, H3K4me3, H3K9me3, H3K27ac, H3K27me3, H3K36me3) and chromatin accessibility (DNase-seq and ATAC-seq). We downloaded the DNase-seq, ATAC-seq, and ChIP-seq profiles of the above CREs from ENCODE database. In cases where data were available only in GRCh38 (hg38) assembly, we utilized LiftOver, a tool on the UCSC Genome Browser, to convert the data into the hg19 assembly. For features with continuous values, the annotation for a CpG is the value of the features at the genomic location of the CpG site; for features with binary values, the feature status (0/1) indicates whether the CpG site is within the epigenomic feature or not. For example, TFBSs were included as binary variables indicating the presence of a co-localized ChIP-seq peak.

We classified the TF ChIP-seq, histone ChIP-seq, DNase-seq, and ATAC-seq data of CREs, and obtained the union of the narrow peak files from multiple samples for each tissue (S1 Data). Transverse colon, ovary, prostate gland, and testis all had matching data available on ENCODE. However, partial CREs data for kidney, lung, and skeletal muscle were absent on ENCODE. Specifically, kidney lacked the ChIP-seq data for POLR2A, lung lacked the ChIP-seq data for POLR2A and CTCF, and skeletal muscle lacked the DNase-seq, ATAC-seq, and ChIP-seq data for POLR2A and CTCF. Missing epigenomic features were encoded as 0, representing the absence of available signal. To assess the robustness of this approach, we performed a sensitivity analysis by alternatively imputing missing values using the mean or median of each feature calculated across all samples with available measurements (S7 Fig).

### Design of DeepMethylation

DeepMethylation consists of three main modules: a sequence module, an epigenomic module, and an integration module. The model takes a one-hot encoded DNA sequence around the CpG site and 25 epigenomic features annotated for the CpG site as input, and the binary DNA methylation state of CpG site centered in the DNA sequences as output. Details of our model architecture and codes are available in GitHub (https://github.com/wrli1215/DeepMethylation). Below is the detailed description of each module.

The sequence module is based on convolutional neural networks (CNN), which includes four convolutional layers that enables the extraction of local DNA sequence features. The first layer is primarily employed to extract single nitrogenous base and DNA motif information, while the subsequent layers obtain higher-level sequence feature information. We designed the size of the first convolutional kernel based on the common DNA motif length. After evaluating different motif lengths, we selected a DNA motif with 15 base pairs, as this length generated the best prediction performance (S2 Table). Therefore, the input of the sequence module is one-hot encoded 414 bp DNA sequence information (without counting the target CpG site and including each 207 bp DNA fragment upstream and downstream). The input sequence length (414 bp; ± 207 bp around the target CpG) was selected based on prior work (e.g., MRCNN using ~400 bp) and slightly extended to account for edge effects in convolutional operations, ensuring sufficient effective sequence context for feature extraction. For the input matrix s_n,x,y_,


$$\begin{array}{c} L_{n,\text{1}}=\;\sum_{x=\text{1}}^{\text{4}\text{1}\text{4}}\sum_{y=\text{1}}^{\text{4}}s_{n,x,y}\overset{f,\text{1}}{\underset{x,y}{w}}+b^{f,\text{1}} \end{array}$$ (1)

Here, $\overset{f,\text{1}}{\underset{x,y}{w}}$ is the parameter or weight of the convolutional filter f for this layer, and $b^{f,\text{1}}$ is the corresponding bias. Then, the output of the first layer $L_{n,\text{1}}$ for each CpG site is a 400*1 tensor with 16 channels. To extract the information contained in the DNA sequence pattern, the output tensor is reshaped into a 20*20 tensor before being input into the next layer. The second layer is the traditional convolution and pooling layer. The size of the convolutional kernel is 3*3, the pooling method is max pooling, and the step sizes are 1*1 and 3*3. Through this layer, high-level sequence features can be extracted.


$$\begin{array}{c} L_{n,\text{2}-\text{1}}=ReLU(\;\sum_{x=\text{1}}^{\text{2}\text{0}}\sum_{y=\text{1}}^{\text{2}\text{0}}L_{n,\text{1}}\overset{f,\text{2}}{\underset{x,y}{w}}+\;b^{f,\text{2}}\;) \end{array}$$ (2)

$$\begin{array}{c} L_{n,\text{2}-\text{2}}=\;{max}_{\text{3}i\leq x,\;\text{3}i\leq y}(L_{i,n,\text{2}-\text{1}}) \end{array}$$ (3)

The rectifier operation (ReLU) activation function sets negative values to zero, such that $L_{n,\text{2}-\text{1}}$ corresponds to the evidence that the motif represented by $\overset{f,\text{2}}{\underset{x,y}{w}}$ occurs at the corresponding position. Nonoverlapping pooling is implemented to decrease the dimensions of the input tensors and, hence, the number of model parameters. Following the first convolutional layer, the next two layers are both single-convolution layers with the same size and step size as the second layer’s convolution kernel. The convolution of all layers is linear, with no pooling layer connection or activation function. Finally, the tensor obtained by the last layer is expanded through the fully connected layer.

The epigenomic module is built using multilayer perceptron (MLP), which captures the epigenomic information. This module contains a single hidden layer with a ReLU activation function to propagate positive outputs and eliminate negative outputs. The input to this module is a vector of 25 lengths. The features learned by the sequence module and the epigenomic module are concatenated with a fully connected layer. To prevent overfitting, a dropout layer with a dropout rate of 0.5 is applied, followed by a softmax layer to predict DNA methylation status at CpG sites in sample of the same tissue.

### Model training and evaluation

We split the CpGs into three independent sets by chromosome for model training and evaluation. Specifically, we used CpG sites on chromosomes 1–11 as the training set, comprising a total of 474,851 CpG sites. For model tuning, we used CpG sites on chromosomes 12–16 as the validation set, which included 144,234 CpG sites. The remaining CpG sites on chromosomes 17–22 constituted the testing set, consisting of 135,034 CpG sites, and were used for evaluating prediction performance.

DeepMethylation was trained independently for each sample, with the final model evaluation results derived by averaging the outcomes across samples of the same tissue. We quantified the performance of the results using the accuracy (ACC), specificity (SP), sensitivity (SE), precision, and F1 score. Note that the functional CpG sites were those that were hypomethylated. The evaluation indicators were calculated as follows:


$$\begin{array}{c} ACC\;=\;\frac{TP\;+\;TN}{TP\;+\;FP\;+\;TN\;+FN} \end{array}$$ (4)

$$\begin{array}{c} SP\;=\;\frac{TN}{TN\;+\;FP} \end{array}$$ (5)

$$\begin{array}{c} SE\;=\;Recall\;=\;\frac{TP}{TP\;+\;FN} \end{array}$$ (6)

$$\begin{array}{c} Precision\;=\;\frac{TP}{TP\;+\;FP} \end{array}$$ (7)

$$\begin{array}{c} F\text{1}\;=\frac{\text{2}\;\times \text{ Precision }\times \text{ Recall}}{\text{Precision }+\text{ Recall}} \end{array}$$ (8)

where true positives (*TP*), true negatives (*TN*), false positives (*FP*), false negatives (*FN*) were derived based on the prediction results. We also computed area under the receiver operating characteristic curve (AUROC) and area under the precision-recall curve (AUPRC), which reflected the overall prediction performance considering both type I (*FPs*) and type II errors (*FNs*).

### Comparison with the state-of-the-art models

We compared the performance of our DeepMethylation model with that of the state-of-the-art model, MRCNN [27], in predicting DNA methylation status. MRCNN was a CNN-based model that captured local sequence information to predict methylation status. However, MRCNN failed in considering the tissue-specificity of the methylation data and did not have the ability to handle the epigenomic features. In addition, we compared our model with two previously established DNA methylation prediction models [12,43], which are respectively based on Random Forest (RF), an ensemble method built on multiple decision trees, and eXtreme Gradient Boosting (XGBoost), a boosting framework that optimizes arbitrary differentiable loss functions. The input of these models was the same as that of DeepMethylation. All models were evaluated in the same testing set for a fair comparison.

### External validation on WGBS data

The whole-genome bisulfite sequencing (WGBS) data was downloaded from NCBI Gene Expression Omnibus (GEO: GSE186458) [44]. We selected WGBS data aligned to the hg19 genome in blood tissue, containing 36 samples of 14 primary cell types. Only CpG sites with greater than 10 × coverage was included. DNA methylation level data from the X and Y chromosomes were excluded. After data processing and quality control, we finally collected an average of ~24 million CpG sites in each sample, with the average coverage ranging from 15.2 to 38.6 reads per CpG site.

We utilized the trained blood-specific DeepMethylation model to predict the DNA methylation status of CpG sites in WGBS data. The whole WGBS data set was used as the external testing set to evaluate the model trained on the EPIC array. We categorized each CpG site in a WGBS sample based on whether that CpG site was assayed on the EPIC array (EPIC site) or not (non-EPIC site) to further evaluate the performance of DeepMethylation on whole-genome prediction.

### Regression-based prediction of continuous methylation levels

To further evaluate whether DeepMethylation can capture continuous DNA methylation variation, we performed regression-based analyses using the same model architecture. In this setting, the output layer was modified to predict continuous methylation beta values instead of binary states. Specifically, the sigmoid activation function was replaced with a linear activation, and the model was trained using a mean squared error loss function. The regression model was first trained and evaluated on the same EPIC array dataset used for the binary classification task, using the same input features and training procedures as the binary model, thereby enabling a direct comparison between classification and regression formulations. Model performance was assessed by comparing predicted and observed methylation levels using the Pearson correlation coefficient (PCC), coefficient of determination (R^2^), mean absolute error (MAE), and mean squared error (MSE). To further assess genome-wide performance, the EPIC-trained regression model was applied to WGBS data, and agreement between predicted and observed methylation levels was evaluated using the same metrics.

### Imputation of 450k data to EPIC data

DNA methylation data of the Illumina Infinium HumanMethylation450 BeadChip (450k array) in GM12878 cell line were obtained from the ENCODE database (accession: ENCSR000ACX). Data preprocessing and quality control were performed using the Minfi R package, resulting in 435,784 high-quality CpG sites retained for downstream analysis. The 450k dataset was used to train the DeepMethylation model, and its performance was evaluated using EPIC array data from blood tissue samples. To further assess the model’s generalizability in imputing DNA methylation levels from the 450k to the EPIC platform, CpG sites were categorized into two groups: those shared by both arrays (450k sites) and those unique to the EPIC array (non-450k sites). Model performance was then independently evaluated within each group to examine its accuracy on both overlapping and novel CpG sites.

### Cross-tissue performance

We utilized the previously processed EPIC methylation data from the nine distinct tissue types (as described in the Data collection and preprocessing section) to assess the model’s cross-tissue generalizability. For each tissue, a dedicated DeepMethylation model was trained exclusively on its corresponding samples. Each tissue-specific model was then evaluated across all nine tissue types, generating 81 unique training-testing tissue combinations. Performance metrics (AUROC/AUPRC) were calculated by averaging predictions across all samples within each tissue pair, with standard deviation computed to quantify variability.

### Delta Methylation model

We designed a variant-evaluation model, DDM, to predict the genetic effect of SNPs on the DNA methylation level of CpG sites. Briefly, we first introduced a variant allele in a given DNA sequence of 207 bp flanking a CpG site. Each sequence (with or without the introduced variant allele) was then passed to the trained DeepMethylation model to get the predicted DNA methylation levels of the CpG sites centered within each sequence. The impact of the introduced variant on DNA methylation level of the CpG site was estimated by the difference of the predicted DNA methylation probability of the CpG site within the two sequences. We did this analysis for 7,156,800 SNPs observed in East Asians generated by our previous study [38], resulting in estimated effects on DNA methylation levels for SNP-CpG pairs in each sample used for training the DeepMethylation model. SNPs were ranked in descending order by their absolute values of predicted effects for each paired CpG site in each sample. The relative effect signal was measured by dividing the absolute value of predicted genetic effect of each SNP by the maximum value of top SNP for each CpG site. The relative signal of the association analysis was calculated by taking the logarithm of the absolute value of the T-value in the same manner. The LD of each SNP was estimated by PLINK [45].

### Evaluation of concordance with mQTL signals

To evaluate the concordance between DDM-predicted variant effects and mQTL-derived signals, we performed several complementary analyses. We first examined the relationship between the distance of SNPs to CpG sites and their predicted effect sizes, and compared this pattern with that observed in mQTL analyses. We then assessed indirect concordance by testing whether SNP-CpG pairs with larger predicted effect sizes were associated with stronger mQTL signals. For this analysis, SNP-CpG pairs within a 414-bp window of EPIC array CpG sites were considered, and mQTL association strength was quantified using the absolute value of the test statistics. To directly compare predicted and empirical effects, we next compared DDM-predicted effect sizes (Δ methylation) with mQTL-derived effect estimates for matched SNP-CpG pairs. Concordance was assessed in terms of both effect size trend and directional consistency. Finally, to assess the potential influence of linkage disequilibrium (LD), we examined the relationship between predicted effect sizes and LD strength relative to the lead SNP at each locus. LD was quantified using pairwise correlation (r^2^), and the dependence of both mQTL association strength and DDM-predicted effects on LD was compared.

### Definition of co-methylation patterns

When neighboring CpG sites are located within a specific distance (D) both upstream and downstream of the target CpG site, target CpG sites are divided into consistent and inconsistent patterns, according to the consistency of the DNA methylation status between neighboring CpG sites and the target CpG sites. If the target CpG site has no neighboring CpG sites within the specific distance (D) upstream and downstream, it is classified as a distal pattern. In this study, the specific disitance, D, is set to 207 bp. This distance threshold was chosen to match the input sequence window of the DeepMethylation model (±207 bp), ensuring that co-methylation patterns were defined within the region where sequence context is explicitly modeled.

## Discussion

In this study, we developed DeepMethylation, a deep learning framework that integrates local DNA sequences and tissue-specific epigenomic annotations to predict DNA methylation status at single-CpG resolution. Our results demonstrate that DeepMethylation outperforms existing methods in both accuracy and AUROC across a range of evaluation scenarios, including array-based, genome-wide, and cross-platform predictions. Importantly, DeepMethylation effectively generalizes to predict methylation patterns beyond the EPIC array coverage and across distinct tissues, supporting its robustness and broad applicability in diverse biological contexts.

Comparing with existing machine learning models, a key innovation of our approach lies in the integration of both sequence context and epigenomic features, which captures complementary information underlying DNA methylation regulation. The ablation analyses revealed that epigenomic annotations contributed more to predictive power than sequence information alone, highlighting the importance of tissue-specific regulatory landscapes in shaping methylation patterns. Through SHAP analysis, we further identified biologically meaningful features—including CpG island context, chromatin accessibility, and promoter regions—as major determinants of methylation state, consistent with known methylation biology. Besides, the DNA methylation data utilized in our study was large-scale and of high quality. After QC, our EPIC array data contained ~750,000 CpG sites in 555 samples across 9 tissues, and WGBS data compromised ~24 million CpG sites in 36 samples representing 14 primary cell types. This scale of data was unprecedented in previous studies, highlighting the robustness and comprehensiveness of our analysis.

Beyond prediction, DeepMethylation also provides insights into the regulatory effects of genetic variants on DNA methylation. By incorporating allele-specific inputs, our variant scoring module (DDM) quantifies the regulatory impact of SNPs on CpG methylation. DDM-predicted variant effects show association with empirical mQTL signals and appear less influenced by LD structure, suggesting that DDM may serve as a useful complement to traditional association-based approaches that can be influenced by genetic linkage. Compared with traditional mQTL analyses, which are often confounded by LD, DDM provides an LD-independent estimate of the potential regulatory impact of individual variants. This framework may improve the prioritization of candidate regulatory variants and serve as a complementary tool alongside association-based approaches such as colocalization, Mendelian randomization, and imputation-driven methylome-wide association studies. To further demonstrate its utility, we applied DDM to predict SNP effects on DNA methylation in blood tissue and made the results publicly available via GitHub (https://github.com/wrli1215/DeepMethylation). This resource provides a computational framework for interpreting the epigenetic impact of genetic variants and facilitates downstream functional studies.

While the binary formulation provides a robust framework for large-scale methylation prediction, it represents a simplification of the underlying biology. DNA methylation is inherently continuous, and intermediate methylation levels are important for capturing cellular heterogeneity, allele-specific methylation, and dynamic changes in gene regulation. To further evaluate this aspect, we implemented a regression-based version of the model using the same EPIC array dataset and found that the framework can be extended to predict continuous methylation levels. In additional evaluation on WGBS data, performance was more modest, highlighting the greater difficulty of continuous prediction beyond array-covered CpG sites. In the context of variant effect prediction, the current DDM framework quantifies genetic effects as changes in predicted methylation probability. Although this does not directly correspond to absolute changes in methylation levels, it reflects the change in the likelihood of a CpG site being methylated given sequence perturbations. Similar probabilistic formulations have been widely used in sequence-based regulatory models (e.g., DeepSEA). We acknowledge that regression-based modeling provides a more direct and biologically interpretable measure of variant effects. At the same time, continuous methylation prediction is more challenging because it is more sensitive to measurement noise and must capture a broader range of quantitative variation across heterogeneous samples. Future work will therefore extend this framework to incorporate continuous methylation prediction more systematically.

The reduced performance observed in genome-wide prediction compared to array-based CpG sites likely reflects fundamental differences in genomic context and CpG distribution between EPIC and non-EPIC CpG sites. EPIC CpG sites are preferentially located in CpG islands, promoter regions, and regulatory elements, whereas genome-wide CpG sites include a larger proportion of CpG-poor and intergenic regions with weaker regulatory signals. These differences introduce a distributional shift between training and evaluation data and highlight the inherent challenges of extending array-based models to the full methylome. Consistent with this interpretation, EPIC and non-EPIC CpG sites show distinct genomic and functional feature distributions (S8–S9 Figs). These findings suggest that, although DeepMethylation can generalize beyond array-covered CpG sites, genome-wide predictions should be interpreted with appropriate caution.

Beyond these considerations, several additional limitations should also be considered. First, due to the use of 414 bp input sequences, the model only captures sequence context within 207 bp of each CpG site, limiting its ability to detect regulatory influences from more distal genomic regions. Future models incorporating longer input sequences may overcome this constraint. Second, the current framework treats epigenomic features as fixed inputs and does not explicitly model genotype-dependent changes in regulatory features. Although our sensitivity analysis suggests that variant effect predictions are robust to moderate perturbations in epigenomic inputs (S3 Text), the true impact of genetic variation on epigenomic states is likely context-specific and remains difficult to quantify at scale. Future work incorporating genotype-aware or allele-specific epigenomic features may further improve model interpretability. Third, our model does not explicitly model neighboring CpG sites. Although incorporating features from the nearest CpG provided only marginal improvement (S1 Text and S1 Fig), the contribution of multiple neighboring CpGs remains unclear under the current architecture. This is constrained by the fixed input structure of the current model, and future work will explore more flexible architectures to capture regional methylation dependencies.

## Conclusion

In conclusion, we present DeepMethylation, a deep learning framework for predicting CpG methylation by integrating local DNA sequence context with tissue-specific epigenomic features. The model achieves strong performance across multiple tissues and prediction settings, including array-based and cross-platform applications, and can generalize beyond EPIC array coverage, although prediction at genome-wide scale remains more challenging. Through feature importance analysis, DeepMethylation provides interpretable insights into the relative contributions of epigenomic factors. The accompanying DDM module provides an LD-independent estimate of the potential regulatory impact of genetic variants on methylation, offering a complementary framework to traditional mQTL analyses for variant prioritization. Collectively, these advances position DeepMethylation as a scalable framework for studying tissue-specific DNA methylation landscapes and prioritizing noncoding variants with potential regulatory relevance.

## Supporting information

S1 Text Contribution of neighboring CpG sites to methylation prediction. (PDF)

S2 Text Comparison with sequence-based variant effect prediction models. (PDF)

S3 Text Sensitivity analysis of variant effect prediction to perturbations in epigenomic features. (PDF)

S1 Fig Effect of incorporating neighboring CpG epigenomic features on model performance. Comparison of predictive performance between the original DeepMethylation model and the model augmented with epigenomic features from the nearest neighboring CpG site. Performance is evaluated using ACC, AUROC, AUPRC, and F1 score. Incorporating neighboring CpG information leads to only marginal improvements across all metrics, indicating limited additional benefit under the current framework.
(TIF)

S2 Fig Regression-based prediction of DNA methylation levels on EPIC array data. Scatter plots comparing predicted and observed DNA methylation levels (beta values) on the EPIC dataset for (A) DeepMethylation and (B) MRCNN. Each point represents a CpG site. The red line indicates the fitted linear regression. Mean absolute error (MAE), mean squared error (MSE), and coefficient of determination (R²) are shown for each model. DeepMethylation shows improved agreement with observed methylation levels compared to MRCNN, as reflected by lower error and higher R² values.
(TIF)

S3 Fig Regression-based evaluation of DNA methylation prediction using WGBS data. Scatter plots comparing predicted and observed DNA methylation levels (beta values) based on WGBS data for (A) all CpG sites, (B) EPIC-covered CpG sites, and (C) non-EPIC CpG sites. Each point represents a CpG site. The red line indicates the fitted linear regression. Mean absolute error (MAE), mean squared error (MSE), and coefficient of determination (R²) are shown for each panel.
(TIF)

S4 Fig Concordance between DDM-predicted effects and mQTL-derived effect estimates. Scatter plot comparing DDM-predicted effect sizes with mQTL-derived effect estimates (beta values) for matched SNP–CpG pairs. Each point represents a SNP–CpG pair, and the red dashed line indicates the fitted linear regression. The mQTL effect was represented by the corresponding beta estimate from association analysis reported previously. This analysis was used to assess both effect size trend and directional consistency between DDM-predicted and empirical variant effects.
(TIF)

S5 Fig Comparison of DDM and DeepSEA-based models in predicting mQTL effect direction. Receiver operating characteristic (ROC) curves comparing the performance of the full DDM model, a sequence-only version of DDM (DDM_seq), and three DeepSEA-based models (Beluga_max_abs, Beluga_mean_abs, and Beluga_top*k*_mean) in distinguishing mQTL-supported variants from non-associated variants. Positive variants were defined as SNPs showing evidence of mQTL effects, whereas negative variants were defined as SNPs without mQTL support. Beluga scores were aggregated using three strategies: maximum absolute score (Beluga_max_abs), mean absolute score (Beluga_mean_abs), and top-*k* mean score (Beluga_top*k*_mean). Area under the ROC curve (AUROC) is shown in the legend for each model.
(TIF)

S6 Fig Sensitivity of DDM-predicted variant effects to perturbations in epigenomic features. Scatter plots comparing raw predicted effect sizes with perturbed effect sizes under different levels of random perturbation applied to epigenomic features: (A) 1%, (B) 5%, (C) 10%, and (D) 20%. Each point represents a SNP-CpG pair. The red dashed line indicates y = x. Pearson correlation coefficients (PCC) and R2 values are shown for each panel.
(TIF)

S7 Fig Sensitivity analysis of imputation strategies for missing epigenomic features. Comparison of model performance across three imputation strategies (zero, mean, and median) for tissues with missing epigenomic data (kidney, lung, and skeletal muscle). Performance is evaluated using ACC, AUROC, AUPRC, and F1 score. Model performance remains highly consistent across different imputation strategies, indicating robustness to missing feature handling.
(TIF)

S8 Fig Comparison of genomic and functional feature distributions between EPIC and non-EPIC CpG sites. (A) Proportion of CpG sites in CpG island-related regions (island, shore, shelf, and open sea) for EPIC and non-EPIC CpG sites, with corresponding pie chart representations for EPIC (B) and non-EPIC (C). (D) Distribution of CpG sites across gene structure and functional annotations, with pie chart representations for EPIC (E) and non-EPIC (F). (G) Distribution of CpG sites across regulatory features, including transcription factor binding, histone modifications, and chromatin accessibility signals, with pie chart representations for EPIC (H) and non-EPIC (I).
(TIF)

S9 Fig Comparison of CpG density distribution between EPIC and non-EPIC CpG sites across chromosomes. Proportion of CpG sites from EPIC and non-EPIC sets across chromosomes (chr1-chr22).
(TIF)

S1 Table List of Epigenomic features used in prediction. (XLSX)

S2 Table Predicting performance of blood-specific DeepMethylation model by different lengths of DNA input (different lengths of motif). (XLSX)

S3 Table Predicting performance between each tissue pair. (XLSX)

S1 Data TF ChIP-seq, Histione ChIP-seq, ATAC-seq and DNase-seq accessions for each tissue form ENCODE (hg19). (XLSX)

S2 Data List all 36 samples of WGBS data in blood tissue. (XLSX)
