# Supplementary material for: DeepMethylation: A deep learning framework for tissue-specific DNA methylation prediction and functional variant annotation
Source: PLoS Comput Biol. 2026 Jul 1;22(7):e1014476. doi: 10.1371/journal.pcbi.1014476 (PMC13340841; doi:10.1371/journal.pcbi.1014476)
Supplement: S1 Text — (PDF) [file pcbi.1014476.s001.pdf]

## S1 Text. Contribution of neighboring CpG sites to methylation prediction

DNA methylation is known to exhibit regional dependencies, with coordinated regulation among neighboring CpG sites. To assess the potential contribution of neighboring CpG information to model performance, we conducted an additional analysis incorporating epigenomic features from the nearest CpG site into the DeepMethylation framework. Specifically, for each target CpG site, we identified the closest neighboring CpG and included its epigenomic features as additional input features. We restricted the analysis to the nearest CpG site to maintain a consistent input structure, as the number and spatial distribution of CpG sites vary substantially across genomic regions, making it challenging to systematically incorporate multiple neighboring CpGs within the current model architecture.

We observed only marginal improvements in predictive performance (on the order of $10^{-3}$ in AUROC; S1 Fig), suggesting that incorporating epigenomic features from a single neighboring CpG provides limited additional benefit beyond the current model. This may reflect the fact that local sequence context (±207 bp) and region-level epigenomic features already capture much of the relevant regulatory information. However, we note that this analysis is limited to the nearest CpG site. In regions with dense CpG clusters, coordinated regulation may involve multiple neighboring CpGs, and their combined contribution remains unclear. Extending the model to incorporate a variable number of neighboring CpG sites would require more flexible architectures capable of handling non-uniform input structures, which is beyond the scope of the current study.
