# Supplementary material for: DeepMethylation: A deep learning framework for tissue-specific DNA methylation prediction and functional variant annotation
Source: PLoS Comput Biol. 2026 Jul 1;22(7):e1014476. doi: 10.1371/journal.pcbi.1014476 (PMC13340841; doi:10.1371/journal.pcbi.1014476)
Supplement: S2 Text — (PDF) [file pcbi.1014476.s002.pdf]

S2 Text. Comparison with sequence-based variant effect prediction models

To further evaluate the relative performance of DDM and assess the contribution of methylation-specific sequence modeling, we benchmarked both the full DDM model and a sequence-only version of DDM (DDM_seq) against DeepSEA2.0 (Beluga), a representative sequence-based regulatory variant prediction framework. Beluga predicts the regulatory impact of noncoding variants from local DNA sequence by estimating their effects on diverse chromatin features, including transcription factor binding, chromatin accessibility, and histone modifications. Because Beluga does not directly predict CpG-specific methylation effects, it was used here as a general sequence-based baseline.

For comparison, SNPs were classified as positive or negative according to whether they showed evidence of methylation effects based on the corresponding mQTL beta estimates. DDM_seq was constructed by removing the epigenomic module and retaining only the sequence branch, while variant effects were calculated using the same framework as the full DDM model. Beluga scores were obtained for the same set of SNPs, and three aggregation strategies were used to summarize the predicted variant effects across outputs: maximum absolute score, mean absolute score, and top-*k* mean score.

The predictive performance of DDM, DDM_seq, and Beluga-derived scores was evaluated based on their ability to distinguish mQTL-positive from mQTL-negative SNPs using AUROC. As shown in S5 Fig, DDM_seq achieved higher AUROC than all Beluga-based aggregation strategies, indicating improved prioritization of methylation-associated variants through methylation-specific sequence modeling. Incorporation of tissue-specific epigenomic information further improved performance, with the full DDM model achieving the highest AUROC. Although the overall discrimination remained modest, these results support a hierarchical contribution of the model components, where methylation-specific sequence modeling provides the major gain and epigenomic information offers additional predictive value.
