# Supplementary material for: DeepMethylation: A deep learning framework for tissue-specific DNA methylation prediction and functional variant annotation
Source: PLoS Comput Biol. 2026 Jul 1;22(7):e1014476. doi: 10.1371/journal.pcbi.1014476 (PMC13340841; doi:10.1371/journal.pcbi.1014476)
Supplement: S3 Text — (PDF) [file pcbi.1014476.s003.pdf]

S3 Text. Sensitivity analysis of variant effect prediction to perturbations in epigenomic features

To evaluate the potential impact of treating epigenomic features as static inputs in the DDM framework, we performed a sensitivity analysis by introducing controlled perturbations to epigenomic features during variant effect prediction. This analysis was motivated by the fact that genetic variants may influence regulatory features such as chromatin accessibility, transcription factor binding, and histone modifications, which are not explicitly modeled in the current framework. Specifically, for each SNP-CpG pair, we randomly perturbed a subset of epigenomic features at varying levels (1%, 5%, 10%, and 20%) and recomputed the predicted variant effects. Perturbations were applied by randomly modifying selected feature values under a given proportion, while keeping the remaining features unchanged. The perturbed predictions were then compared with the original predictions to assess the stability of variant effect estimation.

We observed that at low perturbation levels (e.g. 1-10%), predicted variant effects remained highly consistent with the original predictions, with most data points closely aligned along the identity line (S6A-6C Fig). This indicates that the model is robust to moderate variations in epigenomic inputs. At higher perturbation levels (e.g. 20%), deviations became more pronounced, particularly for variants with larger predicted effects, although the overall correlation structure was largely preserved (S6D Fig).

These findings suggest that, under realistic levels of epigenomic variation, the DDM framework provides stable estimates of variant effects. However, this analysis is limited by the use of random perturbations, which may not fully capture the complex and context-specific manner in which genetic variants influence epigenomic features in vivo. In practice, the magnitude and direction of genotype-dependent epigenomic changes are difficult to quantify and are not systematically available at scale, making it challenging to incorporate such effects into predictive models.

Therefore, treating epigenomic features as fixed inputs represents a tractable approximation that enables the isolation of sequence-driven regulatory effects, while acknowledging that future models incorporating genotype-aware epigenomic features may further improve biological interpretability.
