# Supplementary figures and images for: DeepMethylation: A deep learning framework for tissue-specific DNA methylation prediction and functional variant annotation
Source: PLoS Comput Biol. 2026 Jul 1;22(7):e1014476. doi: 10.1371/journal.pcbi.1014476 (PMC13340841; doi:10.1371/journal.pcbi.1014476)

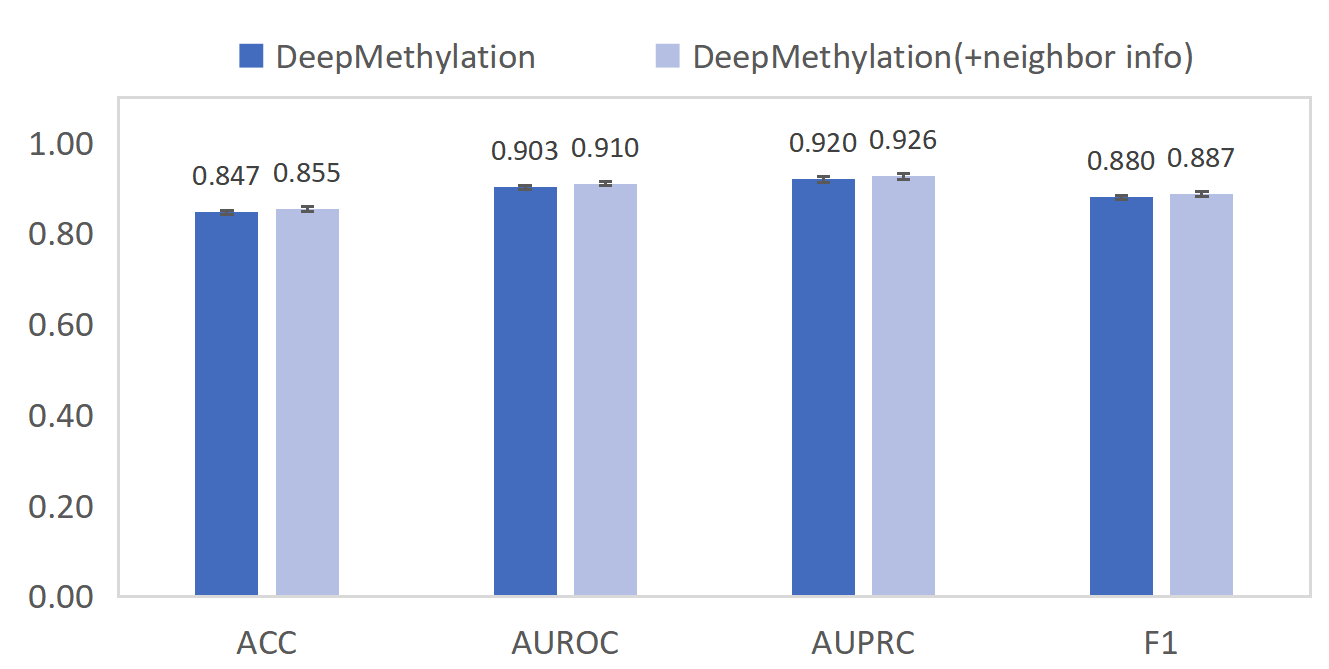

Supplement: S1 Fig — Comparison of predictive performance between the original DeepMethylation model and the model augmented with epigenomic features from the nearest neighboring CpG site. Performance is evaluated using ACC, AUROC, AUPRC, and F1 score. Incorporating neighboring CpG information leads to only marginal improvements across all metrics, indicating limited additional benefit under the current framework. (TIF) [file pcbi.1014476.s004.tif]

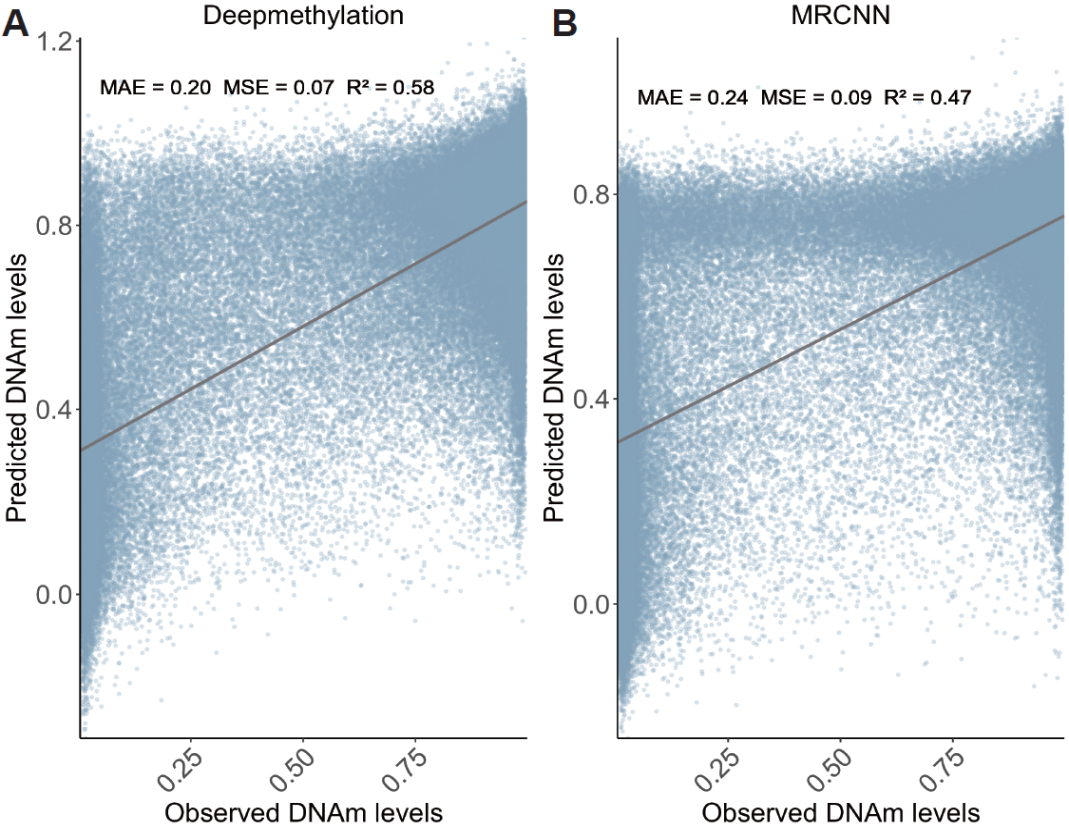

Supplement: S2 Fig — Scatter plots comparing predicted and observed DNA methylation levels (beta values) on the EPIC dataset for (A) DeepMethylation and (B) MRCNN. Each point represents a CpG site. The red line indicates the fitted linear regression. Mean absolute error (MAE), mean squared error (MSE), and coefficient of determination (R²) are shown for each model. DeepMethylation shows improved agreement with observed methylation levels compared to MRCNN, as reflected by lower error and higher R² values. (TIF) [file pcbi.1014476.s005.tif]

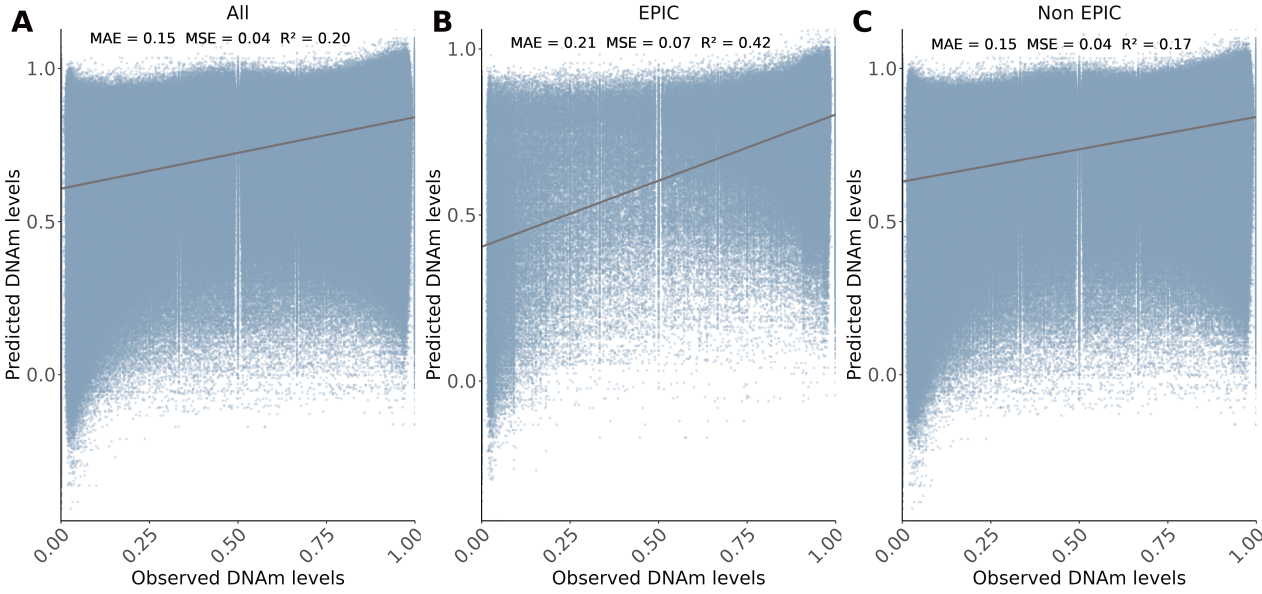

Supplement: S3 Fig — Scatter plots comparing predicted and observed DNA methylation levels (beta values) based on WGBS data for (A) all CpG sites, (B) EPIC-covered CpG sites, and (C) non-EPIC CpG sites. Each point represents a CpG site. The red line indicates the fitted linear regression. Mean absolute error (MAE), mean squared error (MSE), and coefficient of determination (R²) are shown for each panel. (TIF) [file pcbi.1014476.s006.tif]

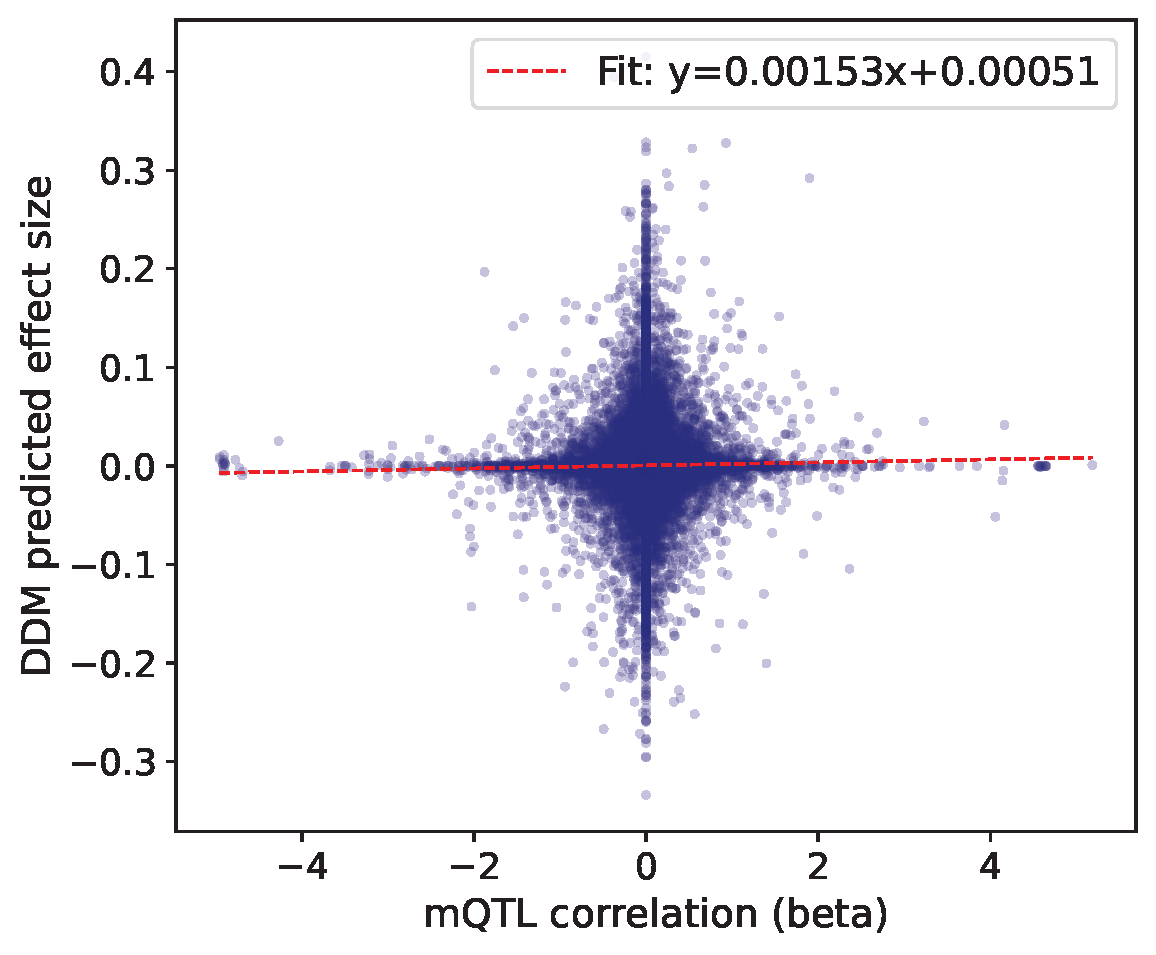

Supplement: S4 Fig — Scatter plot comparing DDM-predicted effect sizes with mQTL-derived effect estimates (beta values) for matched SNP–CpG pairs. Each point represents a SNP–CpG pair, and the red dashed line indicates the fitted linear regression. The mQTL effect was represented by the corresponding beta estimate from association analysis reported previously. This analysis was used to assess both effect size trend and directional consistency between DDM-predicted and empirical variant effects. (TIF) [file pcbi.1014476.s007.tif]

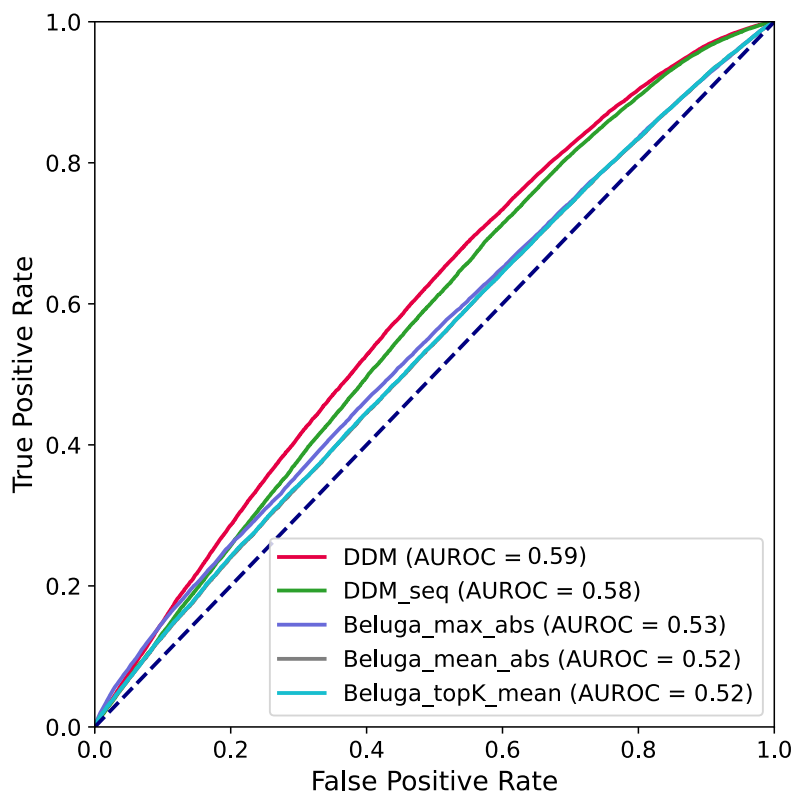

Supplement: S5 Fig — Receiver operating characteristic (ROC) curves comparing the performance of the full DDM model, a sequence-only version of DDM (DDM_seq), and three DeepSEA-based models (Beluga_max_abs, Beluga_mean_abs, and Beluga_topk_mean) in distinguishing mQTL-supported variants from non-associated variants. Positive variants were defined as SNPs showing evidence of mQTL effects, whereas negative variants were defined as SNPs without mQTL support. Beluga scores were aggregated using three strategies: maximum absolute score (Beluga_max_abs), mean absolute score (Beluga_mean_abs), and top-k mean score (Beluga_topk_mean). Area under the ROC curve (AUROC) is shown in the legend for each model. (TIF) [file pcbi.1014476.s008.tif]

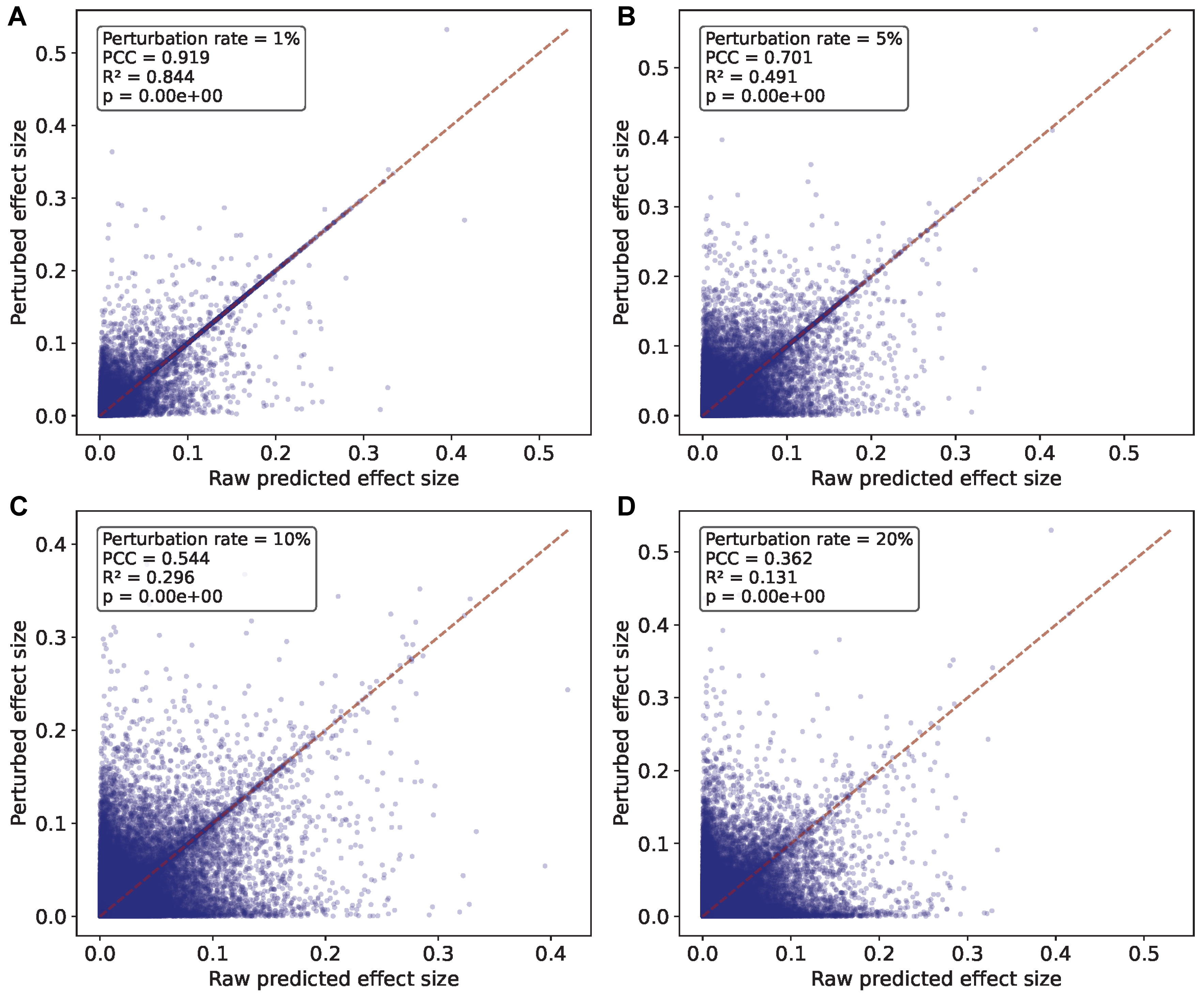

Supplement: S6 Fig — Scatter plots comparing raw predicted effect sizes with perturbed effect sizes under different levels of random perturbation applied to epigenomic features: (A) 1%, (B) 5%, (C) 10%, and (D) 20%. Each point represents a SNP-CpG pair. The red dashed line indicates y = x. Pearson correlation coefficients (PCC) and R2 values are shown for each panel. (TIF) [file pcbi.1014476.s009.tif]

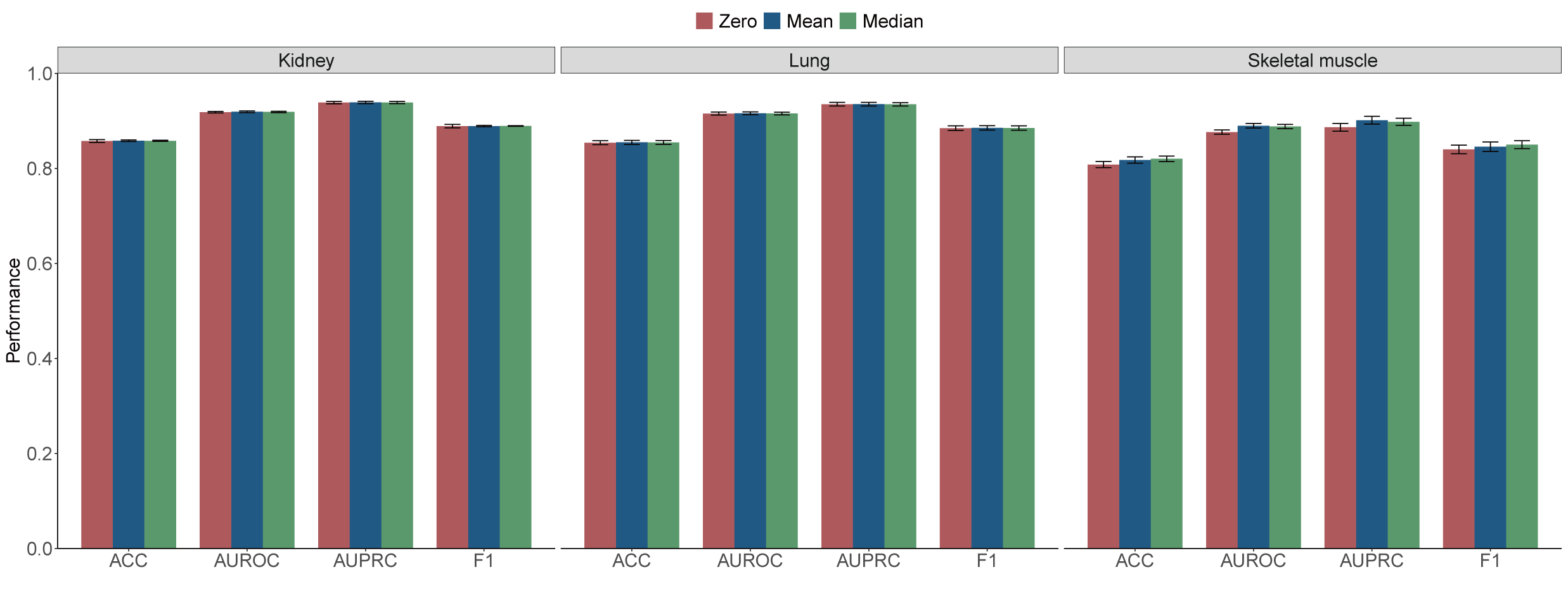

Supplement: S7 Fig — Comparison of model performance across three imputation strategies (zero, mean, and median) for tissues with missing epigenomic data (kidney, lung, and skeletal muscle). Performance is evaluated using ACC, AUROC, AUPRC, and F1 score. Model performance remains highly consistent across different imputation strategies, indicating robustness to missing feature handling. (TIF) [file pcbi.1014476.s010.tif]

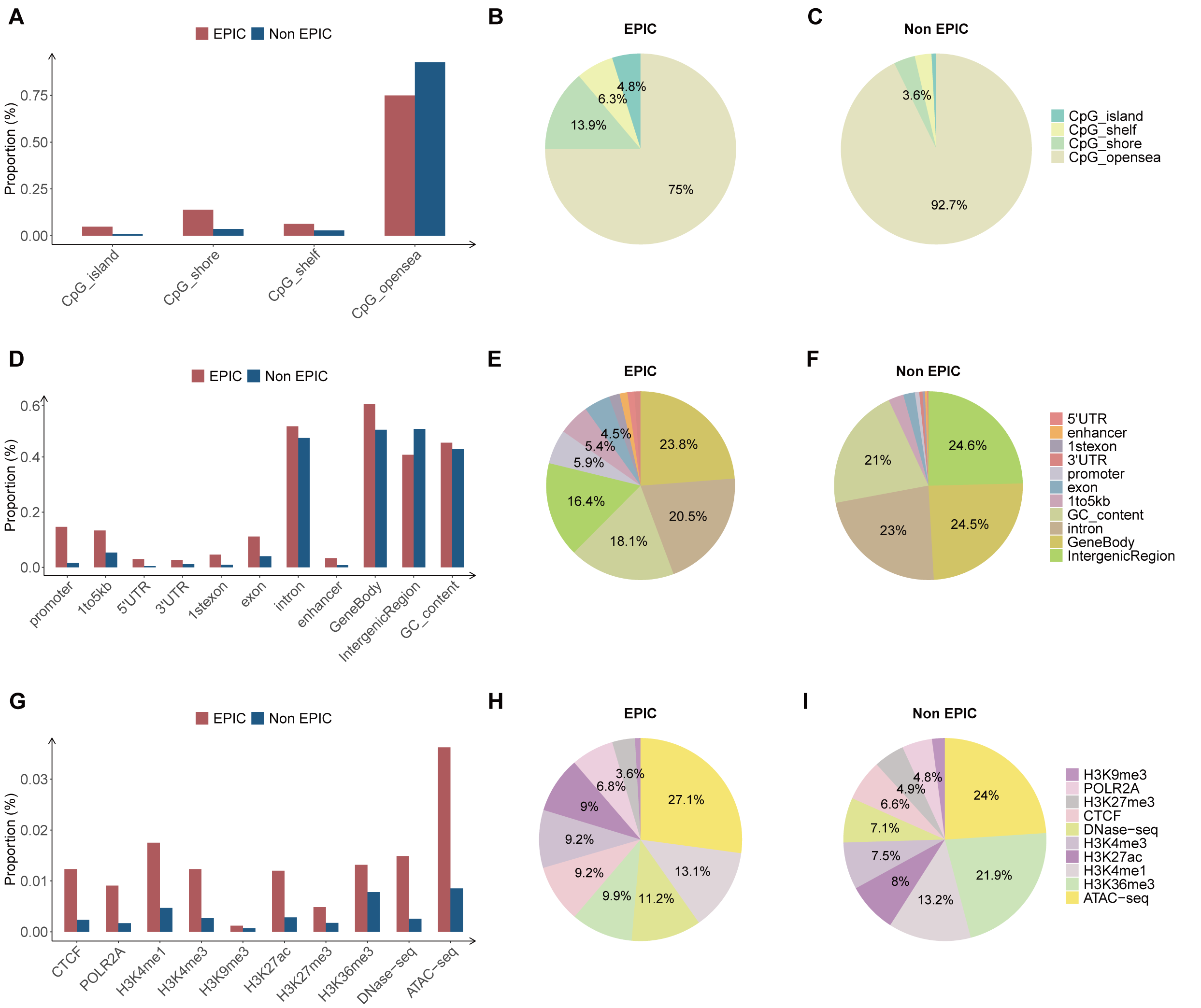

Supplement: S8 Fig — (A) Proportion of CpG sites in CpG island-related regions (island, shore, shelf, and open sea) for EPIC and non-EPIC CpG sites, with corresponding pie chart representations for EPIC (B) and non-EPIC (C). (D) Distribution of CpG sites across gene structure and functional annotations, with pie chart representations for EPIC (E) and non-EPIC (F). (G) Distribution of CpG sites across regulatory features, including transcription factor binding, histone modifications, and chromatin accessibility signals, with pie chart representations for EPIC (H) and non-EPIC (I). (TIF) [file pcbi.1014476.s011.tif]

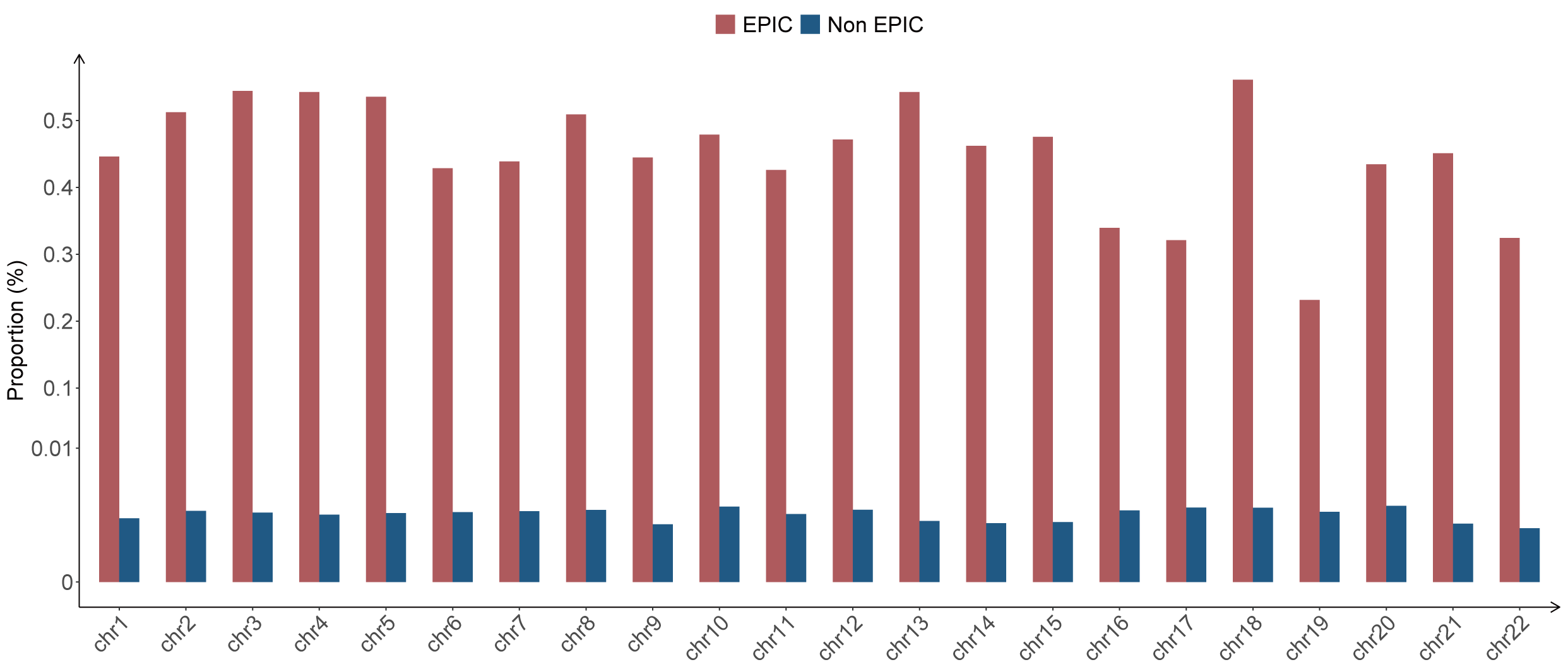

Supplement: S9 Fig — Proportion of CpG sites from EPIC and non-EPIC sets across chromosomes (chr1-chr22). (TIF) [file pcbi.1014476.s012.tif]
